# Preoperative and postoperative nomograms for predicting early recurrence of hepatocellular carcinoma without macrovascular invasion after curative resection

**DOI:** 10.1186/s12893-022-01682-0

**Published:** 2022-06-17

**Authors:** Yanfang Zhang, Xuezhong Lei, Liangliang Xu, Xiaoju Lv, Mingqing Xu, Hong Tang

**Affiliations:** 1 Center of Infectious Diseases, West China Hospital, Sichuan University, Chengdu, China; 2 Department of Liver Surgery and Liver Transplantation Center, West China Hospital, Sichuan University, Chengdu, China

**Keywords:** Hepatocellular carcinoma, Curative liver resection, Early recurrence, Nomogram

## Abstract

**Background:**

Postoperative early recurrence (ER) is a major obstacle to long-term survival after curative liver resection (LR) in patients with hepatocellular carcinoma (HCC). This study aimed to establish preoperative and postoperative nomograms to predict ER in HCC without macrovascular invasion.

**Methods:**

Patients who underwent curative LR for HCC between January 2012 and December 2016 were divided into training and internal prospective validation cohorts. Nomograms were constructed based on independent risk factors derived from the multivariate logistic regression analyses in the training cohort. The predictive performances of the nomograms were validated using the internal prospective validation cohort.

**Results:**

In total, 698 patients fulfilled the eligibility criteria. Among them, 265 of 482 patients (55.0%) in the training cohort and 120 of 216 (55.6%) patients in the validation cohort developed ER. The preoperative risk factors associated with ER were age, alpha-fetoprotein, tumor diameter, and tumor number, and the postoperative risk factors associated with ER were age, tumor diameter, tumor number, microvascular invasion, and differentiation. The pre- and postoperative nomograms based on these factors showed good accuracy, with concordance indices of 0.712 and 0.850 in the training cohort, respectively, and 0.754 and 0.857 in the validation cohort, respectively. The calibration curves showed optimal agreement between the predictions by the nomograms and actual observations. The area under the receiver operating characteristic curves of the pre- and postoperative nomograms were 0.721 and 0.848 in the training cohort, respectively, and 0.754 and 0.844 in the validation cohort, respectively.

**Conclusions:**

The nomograms constructed in this study showed good performance in predicting ER for HCC without macrovascular invasion before and after surgery. These nomograms would be helpful for doctors when determining treatments and selecting patients for regular surveillance or administration of adjuvant therapies.

## Background

Hepatocellular carcinoma (HCC) is the sixth most common cancer and the third leading cause of cancer-related death worldwide [1, 2]. Chronic hepatitis virus infection still is the prominent cause of HCC [3]. Hepatitis B virus (HBV) infection is the leading cause of HCC in Eastern Asian countries and most African countries [4]. Hepatitis C virus (HCV) is the leading virus-related cause of HCC in North America, Europe, Japan, parts of central Asia including Mongolia, and northern Africa and the Middle East, particularly Egypt [5]. And co-infection of hepatitis D virus (HDV) with HBV significantly enhances the recurrence risk of HCC patients after live donor liver transplantation (LT) [6]. Nonalcoholic fatty liver disease (NAFLD) is now the most common liver disease and a major risk factor for HCC in most developed countries [7, 8]. Other risk factors of HCC include alcohol abuse, exposure to dietary toxins such as aflatoxins and aristolochic acid [9]. LT, liver resection (LR), and radiofrequency ablation (RFA) are the three main curative modalities for HCC [2, 10]. Because of a shortage of donor livers and tumor location or diameter limitations, LR is the most common therapy for early and partial-intermediate stage HCC [10]. Remarkable improvements in surgical techniques and perioperative management have allowed selected patients with advanced-stage HCC to also undergo LR [11–14]. Unfortunately, the dramatically high incidence of postoperative recurrence significantly decreases the survival expectancy of patients with HCC after curative LR [2, 15, 16].

There are two common patterns of postoperative recurrence of HCC: early recurrence (ER) (≤ 2 years), which is derived from occult metastasis from the initial tumor, and late recurrence or de novo HCC (> 2 years after surgery), which arises from underlying liver diseases [17–20]. Since ER accounts for up to 70% of all postoperative recurrences and indicates poor long-term survival, it has garnered more attention [18–20]. Multiple risk factors associated with ER have been identified, such as microvascular invasion (MVI), high preoperative alpha fetoprotein (AFP) level, chronic active hepatitis, the absence of a tumor capsule, and large tumor size [17–21]. However, various postoperative therapies such as transcatheter arterial chemoembolization (TACE) [22–24], adoptive immunotherapy [25], iodine-131-labeled lipiodol [26, 27], interferon therapy [28], and cancer vaccines [29] have been shown to delay the postoperative recurrence of HCC, although these results need to be further verified. Therefore, identifying patients at high risk for ER who should receive adjuvant therapies might be a promising avenue for prolonging overall survival after curative LR.

Nomograms for predicting the outcomes of various diseases have been accepted by many investigators [30–34]. A nomogram is constructed based on the independent risk factors of special endpoints and is more accurate than commonly used staging systems [35]. Recently, Zhang et al. [36] established a nomogram to predict the incidence of ER in HCC with portal vein tumor thrombus after R0 LR. However, most curative LRs are performed in patients without macrovascular invasion, and nomograms for predicting ER in this subgroup of patients are lacking. Additionally, to the best of our knowledge, no study has analyzed the relationship between preoperative clinical parameters and ER. We believe that effective models for the prediction of ER would be helpful for surgeons in selecting optimal therapies and designing personalized surveillance strategies for HCC patients, especially during COVID-19 worldwide pandemic [37].

In this study, independent preoperative and postoperative risk factors for ER were identified in a large cohort of patients with HCC without macrovascular invasion. Two nomograms were then generated to preoperatively and postoperatively predict ER based on these factors. The performances of these nomograms were validated using an internal prospective cohort and receiver operating characteristic (ROC) curves.

## Methods

### Patients

To eliminate the heterogeneity in the treatment of HCC from treatment concepts and surgical techniques, this study included only patients who underwent curative-intent resection for HCC between January 2012 and December 2016 at the West China Hospital, Sichuan University. The inclusion criteria were as follows: (1) pathologically diagnosed HCC without lymph node metastasis; (2) absence of tumor thrombus in the major branches of the portal and hepatic veins; (3) initial curative LR, which was defined as the removal of all recognizable tumors with a clear margin; (4) age not less than 18 years; and (5) Child–Pugh class A liver function. The exclusion criteria were as follows: (1) patients with other types of tumors; (2) loss of follow-up within 2 years; (3) poor function of other major organs (heart, lung, and kidney); and (4) incomplete data. Finally, 698 patients who fulfilled our eligibility criteria were included in this study. Among them, 482 patients who underwent curative LR between January 2012 and December 2014 were allocated to the training cohort, and the remaining 216 patients who underwent curative LR between January 2014 and December 2016 were allocated to the internal prospective validation cohort. Detailed information on the two cohorts is presented in Table 1. This study was approved by the ethics committee of West China Hospital, Sichuan University. Written informed consent was obtained from each patient for the data used in the analysis.

**Table 1:** The baseline and clinical characteristics of HCC patients in the training and validation cohorts

Clinical parameters	Training cohort (n = 482)	Validation cohort (n = 216)	P
Gender (male/female)	404 (83.8%)/78 (16.2%)	185 (85.6%)/31 (14.4%)	0.538
Age (> 60/ ≤ 60 years)	369 (76.6%)/113 (23.4%)	162 (75.0%)/54 (25.0%)	0.376
HBsAg (positive/negative)	417 (86.5%)/65 (13.5%)	187 (87.0%)/29 (13.0%)	0.868
HBV-DNA (≥ 10^3^/ < 10^3^ copies/mL)	261 (54.2%)/221 (45.8%)	107 (49.5%)/109 (50.5%)	0.282
HBeAg (positive/negative)	97 (20.1%)/385 (79.9%)	45 (21.0%)/171 (79.0%)	0.323
AFP (< 20/20–400/ > 400 ng/mL)	157 (32.6%)/119 (24.7%)/206 (42.7%)	68 (31.6%)/54 (25.1%)/94 (43.3%)	0.966
NEU (> 3.56/ ≤ 3.56 × 10^9^/L)	228 (47.4%)/254 (52.6%)	99 (46.0%)/117 (54.0%)	0.741
LYM (> 1.1/ ≤ 1.1 × 10^9^/L)	362 (75.1%)/120 (24.9%)	171 (79.1%)/45 (20.9%)	0.255
PLT (> 100/ ≤ 100 × 10^9^/L)	351 (72.8%)/131 (27.2%)	143 (66.2%)/73 (33.8%)	0.076
NLR (> 3/ ≤ 3)	153 (31.8%)/329 (68.2%)	47 (21.9%)/169 (78.1%)	**0.007**
PLR (> 111/ ≤ 111)	180 (37.3%)/302 (62.7%)	59 (27.4%)/157 (72.6%)	**0.011**
TBIL (> 28/ ≤ 28 μmol/L)	15 (3.1%)/467 (96.9%)	8 (3.7%)/208 (96.3%)	0.689
ALT (> 50/ ≤ 50 IU/L)	161 (33.4%)/321 (66.6%)	76 (35.2%)/140 (64.8%)	0.646
AST (> 40/ ≤ 40 IU/L)	216 (44.8%)/266 (55.2%)	101 (46.8%)/115 (53.2%)	0.633
ALB (> 40/ ≤ 40 g/L)	308 (63.9%)/174 (36.1%)	131 (60.6%)/85 (39.4%)	0.411
ALBI grade (1/2/3)	343 (71.2%)/139 (28.8%)/0 (0%)	136 (62.8%)/80 (37.2%)/0 (0%)	**0.025**
GGT (> 60/ ≤ 60 IU/L)	240 (49.8%)/242 (50.2%)	114 (52.8%)/102 (47.2%)	0.466
PT (> 12.8/ ≤ 12.8 s)	119 (24.7%)/363 (75.3%)	39 (17.9%)/177 (82.1%)	0.344
INR (> 1.15/ ≤ 1.15)	101 (21.0%)/381 (79.0%)	50 (23.1%)/166 (76.9%)	0.515
Fib (> 2/ ≤ 2 g/L)	401 (83.2%)/81 (16.8%)	173 (79.9%)/43 (20.1%)	0.338
Tumor diameter (≤ 5/5–10/ > 10 cm)	219 (45.4%)/199 (41.2%)/65 (13.4%)	105 (48.4%)/89 (41.2%)/29 (13.4%)	0.531
Tumor number (1/2/3)	402 (83.4%)/58 (12.0%)/22 (4.6%)	170 (78.6%)/37 (17.2%)/9 (4.2%)	0.284
BCLC stage (A/B)	409 (84.9%)/73 (15.1%)	178 (82.3%)/38 (17.7%)	0.399
Cirrhosis (present/absent)	296 (61.4%)/186 (38.6%)	153 (70.8%)/63 (29.2%)	**0.016**
Differentiation (I + II/III + IV)	279 (57.9%)/203 (42.1%)	123 (56.9%)/93 (43.1%)	0.816
MVI (present/absent)	204 (42.3%)/278 (57.7%)	86 (39.8%)/130 (60.2%)	0.534
Satellite lesion (present/absent)	72 (14.9%)/410 (85.1%)	31 (14.4%)/185 (85.6%)	0.840
Resection (anatomic/non-anatomic)	250 (51.9%)/232 (48.1%)	110 (50.9%)/106 (49.1%)	0.818

Bold numbers indicate statistical significance

SD: standard deviation; HBsAg: hepatitis B surface antigen; HBV-DNA: hepatitis B virus deoxyribonucleic acid; HBeAg: hepatitis B e antigen; AFP: alpha-fetoprotein; NEU: neutrophil; LYM: lymphocyte; PLT: platelet; NLR: neutrophil-to-lymphocyte ratio; PLR: platelet-to-lymphocyte ratio; TBIL: total bilirubin; ALT: alanine transaminase; AST: aspartate aminotransferase; ALB: albumin; ALBI: albumin-bilirubin score; GGT: gamma-glutamyl transpeptidase; PT: prothrombin time; s: second; INR: international normalized ratio; Fib: fibrinogen; BCLC: Barcelona Clinic Liver Cancer staging system; MVI: microvascular invasion

### Surgery

To prepare for surgery, imaging examinations, including contrast-enhanced computed tomography (CT) and/or magnetic resonance imaging (MRI), were performed to evaluate the characteristics of the tumor. Routine blood tests included blood cell analysis, liver/renal/coagulation function tests, hepatitis B virus (HBV)/HCV screening tests, HBV deoxyribonucleic acid (HBV-DNA) measurement, and serum tumor markers, including AFP, carcinoembryonic antigen (CEA), carbohydrate antigen 19–9 (CA199), and CA125. The albumin-bilirubin (ALBI) score was used to evaluate liver function in each patient and was computed using the following formula: − 0.085 × (albumin, g/L) + 0.66 × log (bilirubin, μmol/L). Patients were stratified into three groups according to previously described cutoffs, resulting in three grades: ALBI grade 1 (≤ − 2.60), grade 2 (> − 2.60 to − 1.39), and grade 3 (> − 1.39). Heart and lung functions were primarily evaluated by electrocardiography and chest radiography, and echocardiography and pulmonary function tests were performed if necessary. Before surgery, a multidisciplinary team consultation was routinely performed to design individual treatments for all patients. Surgical decisions were made based on tumor characteristics, reserve liver function, American Society of Anesthesiologists score [38], and the technological feasibility of LR.

All the eligible patients underwent open surgery. A right subcostal incision with midline extension was performed. Intraoperative ultrasonography (US) was routinely performed to identify additional nodules that were not revealed preoperatively and determine a tumor-free margin of at least 1 cm. Anatomic resection was the first option for patients with an ideal tumor location and no obvious liver cirrhosis. To decrease surgical blood loss, intermittent Pringle maneuver was performed at a cycle of 15/5 min of clamp/unclamp time. After removal from the body, tumor specimens were fixed with 4% paraformaldehyde for 15 min and delivered to the histological department for histological examination. Finally, surgical information, including surgery duration, resection type, blood loss, and transfusion, was recorded carefully.

### Follow-up

The postoperative follow-up program was described in our previous study [39]. In brief, all patients were regularly followed up in the first postoperative month, every 3 months for the next 3 years, and every 6 months thereafter. Abdominal US, serum AFP levels, HBV-DNA load, and liver function were routinely examined at each follow-up. Enhanced CT or MRI scans were performed when suspicious lesions were found or when AFP was persistently elevated. If necessary, bone scintigraphy or positron emission tomography was performed to confirm bone or distant metastases. Tumor recurrence was diagnosed based on the typical appearance of a new lesion on at least two radiological examinations with or without elevated AFP levels. Once HCC recurrence was diagnosed, the most appropriate treatments, such as rehepatectomy, RFA, salvage LT, TACE, sorafenib, and best supportive care, were recommended according to the characteristics of the recurrent tumors and liver function.

Recurrence time was defined as the interval between LR and the first diagnosis of recurrence. In line with previous studies, we classified tumors with a recurrence time of no more than 2 years as ER; otherwise, the recurrence pattern was classified as late recurrence [17–20]. Overall survival (OS) was defined as the interval between LR and death or last follow-up. The follow-up was conducted in March 2019.

### Statistical analysis

All statistical analyses were performed using SPSS version 24.0 (IBM SPSS Inc,

Chicago, IL, USA) and R software version 3.5.0 with the rms package (http://www.r-project.org/). In order to make the prediction models easier to use, all quantitative variables in this study were categorized, including age, tumor size, and all blood test results. Categorical variables are expressed as numbers or percentages and were compared using Pearson’s chi-square or Fisher’s exact test. Univariate and stepwise multivariate analyses were performed using logistic regression to identify independent risk factors related to ER in the training cohort. Nomograms for preoperative and postoperative prediction of ER were generated based on the results of multivariate logistic regression analyses. The predictive performances of the nomograms were evaluated using the concordance index (C-index) and calibration curves. Model performance was validated using the internal prospective validation cohort. For the clinical use of the constructed nomograms, the total pre- and postoperative risk scores of each patient were calculated using the nomograms. ROC curve analysis was performed to calculate the optimal cutoff values that were determined by maximizing the Youden index (sensitivity + specificity − 1). The predictive ability of the optimal cutoff values was assessed based on the sensitivity, specificity, predictive values, and likelihood ratios. All analyses were two-tailed, and statistical significance was set at *P* < 0.05.

## Results

### Patient characteristics

The patient characteristics are shown in Table 1. Except for the neutrophil-to-lymphocyte ratio (NLR) (*P* = 0.007), platelet-to-lymphocyte ratio (PLR) (*P* = 0.011), ALBI grade (*P* = 0.025), and presence of cirrhosis (*P* = 0.016), the baseline and clinicopathological data were comparable between the training and validation cohorts. The median follow-up period for all included patients was 36 months (range, 1–78 months). ER was observed in 265 (55.0%) and 120 (55.6%) patients in the training and validation cohorts, respectively.

### Independent predictors of early recurrence

As shown in Table 2, univariate logistic analyses revealed that multiple variables, including sex, age, hepatitis B surface antigen (HBsAg), HBV-DNA, hepatitis B e antigen, AFP, neutrophil count, platelet count, NLR, PLR, aspartate aminotransferase, gamma-glutamyl transpeptidase, tumor diameter, tumor number, Barcelona Clinic Liver Cancer stage, differentiation, MVI, satellite lesions, and resection type, were significantly associated with ER in the training cohort. Subsequent multivariate analyses further revealed that four preoperative risk factors, including age (*P* < 0.001), AFP level (< 20 vs 20–400 ng/mL, *P* = 0.001; < 20 vs > 400 ng/mL, *P* = 0.004), tumor diameter (≤ 5 vs 5–10 cm, *P* < 0.001; ≤ 5 vs > 10 cm, *P* = 0.008), and tumor number (1 vs 2, *P* = 0.019; 1 vs 3, *P* = 0.035). And five postoperative risk factors, including age (OR = 0.981, 95% CI 0.975–0.987, *P* < 0.001), tumor diameter (≤ 5 vs 5–10 cm, *P* < 0.001; ≤ 5 vs > 10 cm, *P* = 0.003), tumor number (1 vs 2, *P* = 0.003; 1 vs 3, *P* = 0.042), differentiation (*P* = 0.025), and MVI (*P* < 0.001), were significantly associated with ER in HCC patients without macrovascular invasion after curative LR (Table 3).

**Table 2:** Univariate logistic analysis on clinical parameters in predicting early recurrence in the training cohort

Clinical parameters	OR (95% CI)	P
Gender (male/female)	1.27 (1.043–1.545)	**0.017**
Age (> 60/ ≤ 60 years)	0.596 (0.480–0.740)	**< 0.001**
HBsAg (positive/negative)	1.262 (1.075–1.482)	**0.004**
HBV-DNA (≥ 10^3^/< 10^3^ copies/mL)	1.529 (1.164–2.010)	**0.002**
HBeAg (positive/negative)	1.771 (1.170–2.681)	**0.007**
AFP		
(< 20/20–400 ng/mL)	1.337 (1.171–1.527)	**< 0.001**
(< 20/ > 400 ng/mL)	1.582(1.277–1.960)	**< 0.001**
NEU (> 3.56/ ≤ 3.56 × 10^9^/L)	1.621 (1.241–2.117)	**< 0.001**
LYM (> 1.1/ ≤ 1.1 × 10^9^/L)	1.221 (0.993–1.502)	0.059
PLT (> 100/ ≤ 100 × 10^9^/L)	1.472 (1.189–1.821)	**< 0.001**
NLR (> 3/ ≤ 3)	2.000 (1.429–2.799)	**< 0.001**
PLR (> 111/ ≤ 111)	1.687 (1.247–2.282)	**0.001**
TB (> 28/ ≤ 28 μmol/L)	2.000 (0.684–5.581)	0.206
ALT (> 50/ ≤ 50 IU/L)	1.368 (1.000–1.870)	**0.05**
AST (> 40/ ≤ 40 IU/L)	1.602 (1.218–2.108)	**0.001**
ALB (> 40/ ≤ 40 g/L)	1.184 (0.947–1.482)	0.139
ALBI grade (1/2/3)	0.781 (0.523–1.168)	0.229
GGT (> 60/ ≤ 60 IU/L)	1.697 (1.306–2.205)	**< 0.001**
PT (> 12.8/ ≤ 12.8 s)	1.164 (0.812–1.668)	0.41
INR (> 1.15/ ≤ 1.15)	1.244 (0.841–1.842)	0.275
Fib (> 2/ ≤ 2 g/L)	1.079 (0.873–1.335)	0.482
Tumor size		
(≤ 5/5–10 cm)	3.279 (2.189–4.910)	**< 0.001**
(≤ 5/ > 10 cm)	2.031 (1.497–2.755)	**< 0.001**
Tumor number		
(1/2)	2.295 (1.262–4.174)	**0.007**
(1/3)	2.069 (1.193–3.588)	**0.010**
BCLC stage (A/B)	3.056 (1.795–5.203)	**< 0.001**
Cirrhosis (present/absent)	1.193 (0.949–1.499)	0.131
Differentiation (I + II/III + IV)	1.819 (1.365–2.426)	**< 0.001**
MVI (present/absent)	2.923 (2.133–4.005)	**< 0.001**
Satellite lesion (present/absent)	2.429 (1.461–4.037)	**0.001**
Resection (anatomic/non-anatomic)	1.475 (1.146–1.899)	**0.003**

Bold numbers indicate statistical significance

OR: odds ratio; CI: confidence interval; HBsAg: hepatitis B surface antigen; HBV-DNA: hepatitis B virus deoxyribonucleic acid; HBeAg: hepatitis B e antigen; AFP: alpha-fetoprotein; NEU: neutrophil; LYM: lymphocyte; PLT: platelet; NLR: neutrophil-to-lymphocyte ratio; PLR: platelet-to-lymphocyte ratio; TBIL: total bilirubin; ALT: alanine transaminase; AST: aspartate aminotransferase; ALB: albumin; ALBI: albumin-bilirubin score; GGT: gamma-glutamyl transpeptidase; PT: prothrombin time; s: second; INR: international normalized ratio; Fib: fibrinogen; BCLC: Barcelona Clinic Liver Cancer staging system; MVI: microvascular invasion

**Table 3:** Multivariate logistic analysis on clinical parameters in predicting early recurrence in the training cohort

Clinical parameters	OR (95% CI)	P
*Preoperative*		
Age (> 60/ ≤ 60 years)	0.984 (0.987–0.990)	< 0.001
AFP		
(< 20/20–400 ng/mL)	1.508 (1.180–1.928)	0.001
(< 20/ > 400 ng/mL)	1.627 (1.171–2.260)	0.004
Tumor diameter		
(≤ 5/5–10 cm)	2.862 (1.753–4.673)	< 0.001
(≤ 5/ > 10 cm)	1.699 (1.148–2.513)	0.008
Tumor number		
(1/2)	2.540 (1.168–5.523)	0.019
(1/3)	2.399 (1.061–5.420)	0.035
*Postoperative*		
Age (> 60/ ≤ 60 years)	0.981 (0.975–0.987)	< 0.001
Tumor diameter (≤ 5/5–10/ > 10 cm)		
(≤ 5/5–10 cm)	2.835 (1.823–4.408)	< 0.001
(≤ 5/ > 10 cm)	1.717 (1.198–2.462)	0.003
Tumor number (1/2/3)		
(1/2)	1.955 (1.247–3.065)	0.003
(1/3)	2.084 (1.026–4.232)	0.042
Differentiation (I + II/III + IV)	1.580 (1.059–2.358)	0.025
MVI (present/absent)	2.904 (1.914–4.405)	< 0.001

OR: odds ratio; CI: confidence interval; AFP: alpha-fetoprotein; MVI: microvascular invasion

### Construction of pre- and postoperative nomograms for predicting early recurrence

**Fig. 1:**
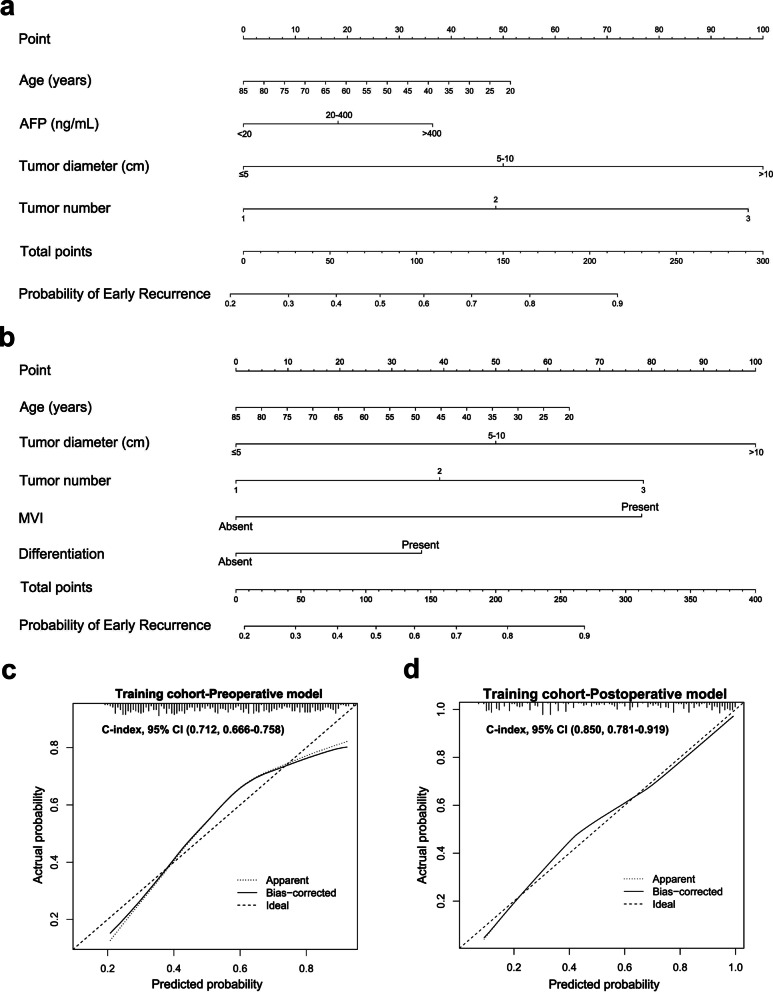
Pre- and postoperative nomograms and calibration curves for predicting early recurrence in training cohort.** a** The preoperative independent risk factors and nomogram for early recurrence. **b** The postoperative independent risk factors and nomogram for early recurrence. **c** The calibration curve of preoperative nomogram in training cohort. **d** The calibration curve of postoperative nomogram in raining cohort

**Fig. 2:**
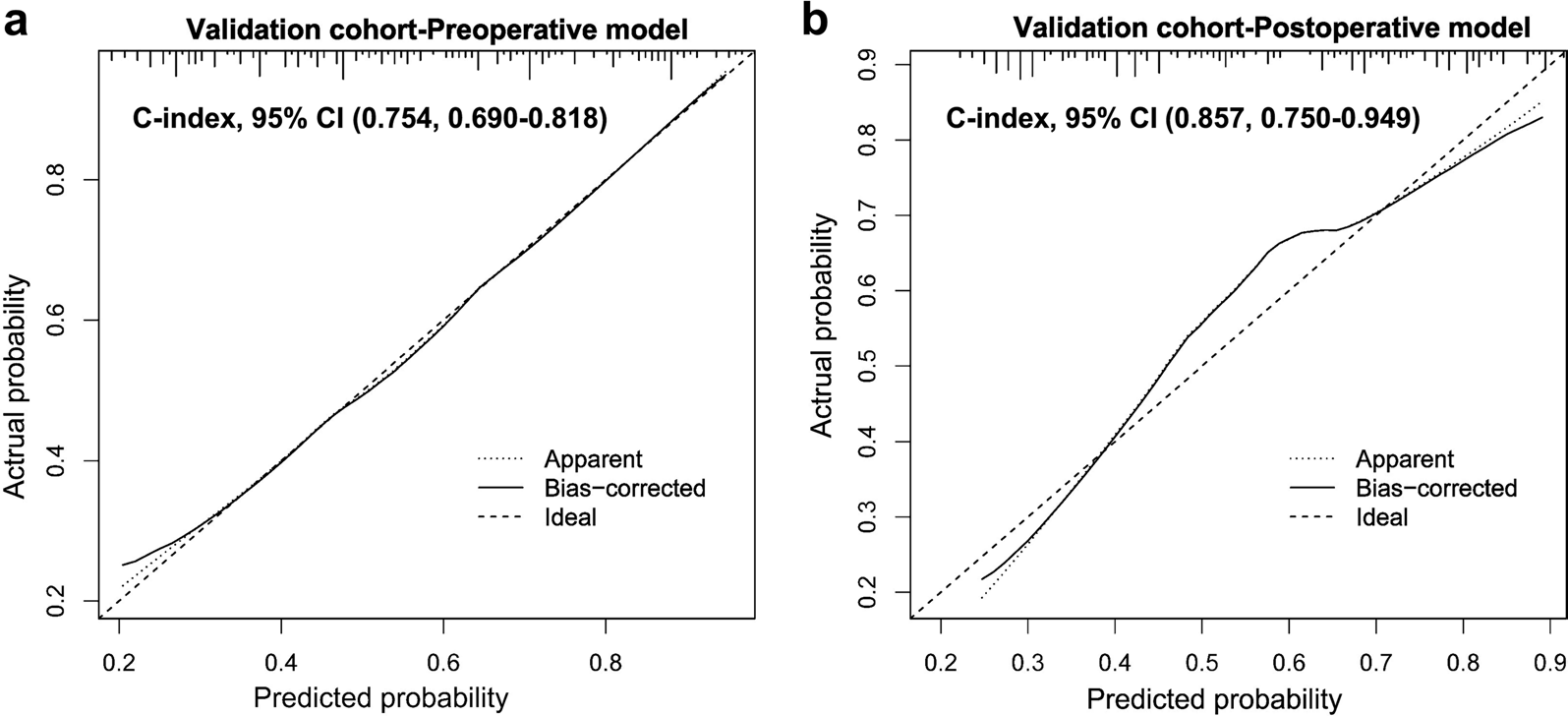
The calibration curves of pre- (**a**) and postoperative (**b**) nomograms in internal prospective validation cohort

Two nomograms that integrated all significant independent factors for pre- and postoperative prediction of ER were generated using the rms package in R (Fig. 2). The C-indices of the pre- and postoperative nomograms in the training cohort were 0.712 (95% CI 0.666–0.758, *P* < 0.001) and 0.850 (95% CI 0.781–0.919, *P* < 0.001), respectively. The calibration plots showed ideal agreement on the incidence of ER between the predictions by the nomograms and the actual observations during follow-up (Fig. 1).

For clinical use of the constructed nomograms, the projection of each variable on the point scale gave a unique score for each variable (Fig. 1). After adding the scores for all variables, the total points for each patient were calculated. Then, the projection of the total points on the probability scale represented the individual probability for ER.

### Validation of the prediction models

The performances of the preoperative and postoperative nomograms were validated using the internal prospective validation cohort. The total preoperative and postoperative points for each patient in the validation cohort were calculated using the two nomograms. Then, the preoperative and postoperative total points were treated as a new risk factor to calculate the C-indices and produce calibration curves for ER. The C-indices for the pre- and postoperative prediction of ER in the validation cohort were 0.754 (95% CI 0.690–0.818, *P* < 0.001) and 0.857 (95% CI 0.750–0.949, *P* < 0.001), respectively. The calibration curves also showed ideal consistency between the predicted and observed probability of ER (Fig. 2).

**Fig. 3:**
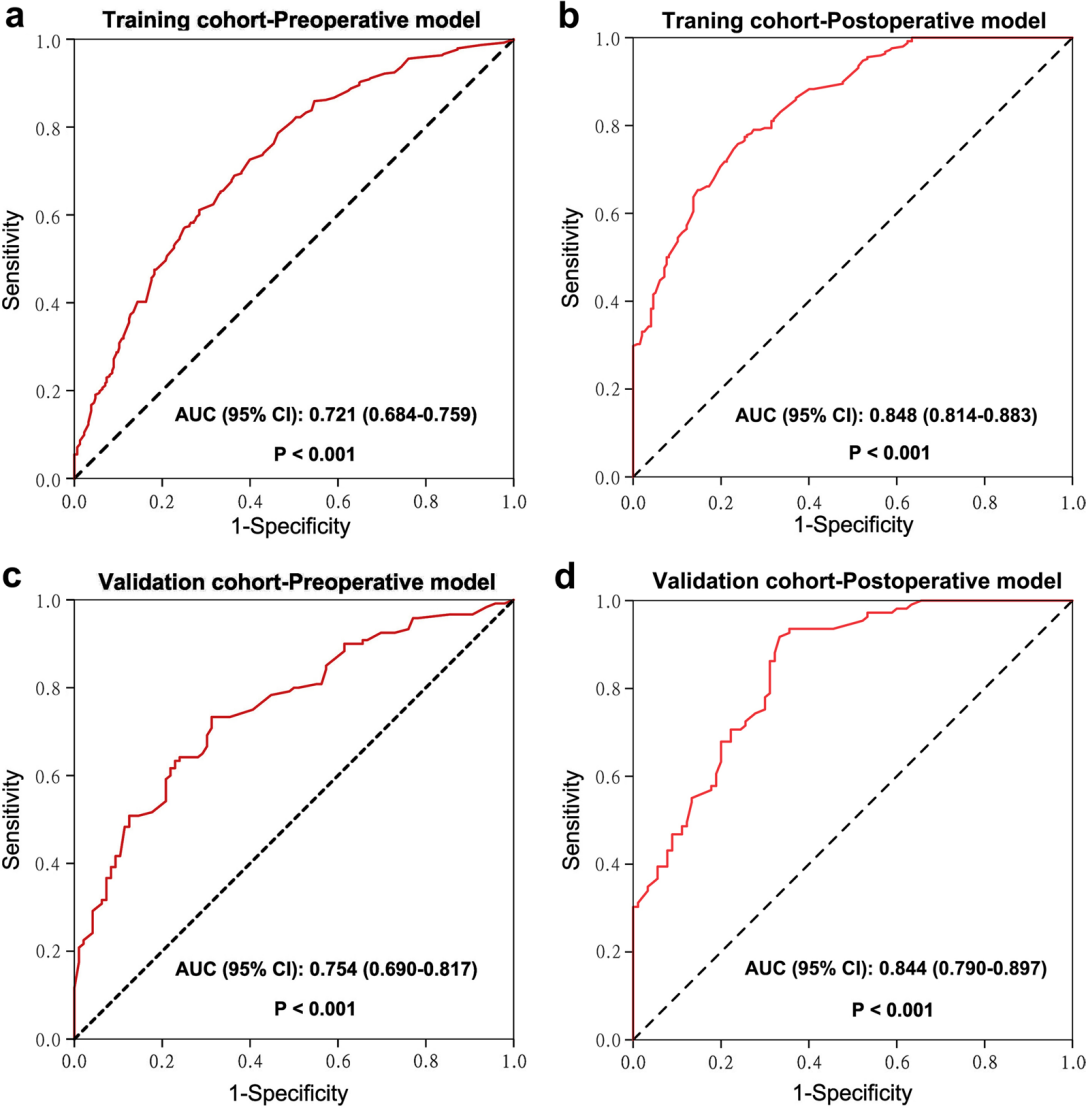
The receiver operating characteristic curves of pre- and postoperative nomograms in training (**a**, **b**) and validation (**c**, **d**) cohorts

In addition, the predictive performances of the nomograms were evaluated using ROC curve analysis (Fig. 3). In the training cohort, the areas under the ROC curves (AUCs) of the pre- and postoperative nomograms were 0.721 (95% CI 0.684–0.759, *P* < 0.001) and 0.848 (95% CI 0.814–0.883, *P* < 0.001) respectively; in the internal prospective validation cohort, the AUCs of the pre- and postoperative nomograms were 0.754 (95% CI 0.690–0.817, *P* < 0.001) and 0.844 (95% CI 0.790–0.897, *P* < 0.001), respectively. These were comparable with the C-indices of the nomograms. These results indicate that the constructed nomograms perform well in predicting ER for patients with HCC without macrovascular invasion after curative LR.

### The predictive ability of the nomograms

The optimal cutoff values of the total preoperative and postoperative nomogram scores for predicting ER were 88 (range: 4–284) and 110 (range: 5–356), respectively (Table 4). For the preoperative model, the sensitivity, specificity, positive predictive value, negative predictive value, positive likelihood ratio, and negative likelihood ratio for distinguishing ER were 0.611, 0.716, 0.704, 0.587, 2.151, and 0.543, respectively, in the training cohort and 0.730, 0.677, 0.733, 0.674, 2.260, and 0.399, respectively, in the validation cohort. For the postoperative model, the sensitivity, specificity, positive predictive value, negative predictive value, positive likelihood ratio, and negative likelihood ratio for distinguishing ER were 0.706, 0.802, 0.793, 0.724, 3.564, and 0.367, respectively, in the training cohort, and 0.679, 0.800, 0.764, 0.699, 3.394, and 0.401, respectively, in the validation cohort.

**Table 4:** Predictive ability of the optimal cut off values on the risk of early recurrence

Variables	Preoperative nomogram		Postoperative nomogram	
	Training cohort (482)	Validation cohort (216)	Training cohort (482)	Validation cohort (216)
AUC	0.721 (0.684–0.759)	0.754 (0.690–0.817)	0.848 (0.814–0.883)	0.844 (0.790–0.897)
Cut-off score	88	88	110	110
Sensitivity	0.611 (0.567–0.654)	0.642 (0.578–0.706)	0.706 (0.665–0.747)	0.679 (0.617–0.741)
Specificity	0.716 (0.676–0.756)	0.76 (0.703–0.817)	0.802 (0.766–0.838)	0.800 (0.746–0.853)
Positive predictive value	0.704 (0.663–0.745)	0.77 (0.714–0.826)	0.793 (0.756–0.829)	0.764 (0.707–0.821)
Negative predictive value	0.587 (0.543–0.631)	0.629 (0.565–0.693)	0.724 (0.684–0.764)	0.699 (0.638–0.760)
Positive likelihood ratio	2.151 (2.011–2.291)	2.675 (2.393–2.957)	3.564 (3.294–3.834)	3.394 (3.014–3.774)
Negative likelihood ratio	0.543 (0.499–0.587)	0.471 (0.404–0.538)	0.367 (0.324–0.411)	0.401 (0.336–0.466)

CI: confidence interval

## Discussion

With the development of surgical techniques and perioperative management, LR has become increasingly safe for early, intermediate, and selected advanced-stage HCCs [40]. Further, prognostic analysis indicates that LR is more effective than other therapies for HCC patients with an advanced tumor burden [41, 42]. However, postoperative recurrence, especially ER, significantly shortens the survival expectancy for patients who undergo curative LR [18, 20, 21]. In addition, repeated treatments after recurrence not only seriously impacted patients’ quality of life but also heavily increased the medical burden.

Predictors of ER have been investigated in numerous studies. Imamura et al. [17] found that non-anatomical resection, the presence of MVI, and serum AFP ≥ 32 ng/mL were significantly associated with ER of HCC after hepatectomy. Portolani et al. [18] reported that cirrhosis, chronic active hepatitis, HCV positivity, vascular infiltration, and transaminase levels were significantly associated with ER in patients with HCC after hepatectomy. Cheng et al. [21] observed that a tumor diameter > 5 cm, the absence of a tumor capsule, and the presence of MVI were correlated with ER of solitary HCC after curative resection. A recent study conducted by Zhang et al. [36] revealed that HBV positivity, advanced portal vein tumor thrombus (PVTT), high HBV-DNA load, the presence of satellite nodules, elevated AFP, and large tumor diameter were significantly associated with ER of HCC with PVTT after R0 LR. In the present study, using a large cohort of HCC patients without macrovascular invasion, four preoperative and five postoperative independent risk factors for ER were identified. The nomograms based on these factors showed good predictive ability for ER, with C-indices of 0.721 and 0.850 for the pre- and postoperative models in the training cohort, respectively, and 0.754 and 0.857 for the pre- and postoperative models in the validation cohort, respectively. Further, the calibration curves in the training and validation cohorts showed ideal agreement between prediction and actual observation.

All risk factors incorporated in the present nomograms are easily obtainable clinically and have been demonstrated to be associated with the prognosis of HCC after curative LR. In this study, age was negatively associated with the incidence of postoperative ER. Compared with younger patients, elderly patients with HCC normally have lower AFP levels, lower rates of HBsAg positivity, and a lower tumor burden [43–45]. Furthermore, the levels of some serum tumor markers are significantly lower in elderly patients than in younger patients [46]. Tumor size and number are commonly used in various HCC staging systems [47–49]. Larger tumor size and more tumors indicate a higher probability of intrahepatic metastasis and a poorer prognosis [50–54]. Serum AFP level is not only a significant prognostic predictor for HCC [54–57], but is also associated with many metastatic characteristics of HCC, such as MVI [58], an incomplete tumor capsule [59, 60], and satellite lesions [61]. MVI is a signal of intrahepatic vessel dissemination [62], and has been repeatedly been shown to be an independent risk factor for ER and poor OS in HCC patients who undergo curative LR [63–65]. Tumors with poor pathological differentiation indicate tumor cells with aggressive behavior, which have a greater ability to proliferate and metastasize than tumor cells with good differentiation [66, 67].

The ROC curves showed that the optimal cutoff values for the pre- and postoperative nomograms were 88 and 110, respectively. A score equal to or greater than the cutoff values indicated a high risk of ER. In clinical practice, the preoperative nomogram might be useful for surgeons when designing therapy for patients with HCC. The postoperative nomogram may serve as a tool for selecting patients for adjuvant therapy and more frequent surveillance.

This study has some limitations. First, the models were constructed based on retrospective data, and their performance needs to be validated prospectively. Second, this study only included patients from a single center, and future external validation is necessary. Third, the main etiology of HCC in the present study was HBV infection, and the performances of the present models for HCC with other etiologies need to be validated. Finally, examination of other recurrence-related factors is necessary to further improve the predictive accuracy of these nomograms.

## Conclusion

The present study revealed that four preoperative and five postoperative clinical variables were significantly associated with ER in patients with HCC without macrovascular invasion after curative LR. Two nomograms based on these predictors showed ideal predictive performance. These prediction models are meaningful for doctors when designing treatments before surgery and selecting patients for regular surveillance and administration of adjuvant therapies after surgery.

## Data Availability

The datasets generated and analyzed during the current study are not publicly available because of patient privacy but are available from the corresponding author upon reasonable request.

## Abbreviations

HCC: Hepatocellular carcinoma
HBV: Hepatitis B virus
HCV: Hepatitis C virus
HDV: Hepatitis D virus
LT: Liver transplantation
LR: Liver resection
RFA: Radiofrequency ablation
ER: Early recurrence
MVI: Microvascular invasion
AFP: Alpha-fetoprotein
CEA: Carcinoembryonic antigen
CA199: Carbohydrate antigen 19–9
CA125: Carbohydrate antigen 125
TACE: Transcatheter arterial chemoembolization
ROC: Receiver operating characteristic
AUC: Area under the ROC curve
CT: Computed tomography
MRI: Magnetic resonance imaging
HBV-DNA: Hepatitis B virus deoxyribonucleic acid
ALBI: Albumin-bilirubin
US: Ultrasonography
OS: Overall survival
C-index: Concordance index
NLR: Neutrophil-to-lymphocyte ratio
PLR: Platelet-to-lymphocyte ratio
HBsAg: Hepatitis B surface antigen
OR: Odds ratio
CI: Confidence interval
PVTT: Portal vein tumor thrombus
